# Factors Influencing Adjuvant Chemotherapy and Trastuzumab Choice in Older Human Epidermal Growth Factor Receptor 2-positive Breast Cancer Patients

**DOI:** 10.7150/jca.39509

**Published:** 2020-02-14

**Authors:** Yan Fang, Zheng Wang, Jiayi Wu, Ou Huang, Jianrong He, Li Zhu, Weiguo Chen, Yafen Li, Xiaosong Chen, Kunwei Shen

**Affiliations:** Comprehensive Breast Health Center, Ruijin Hospital, Shanghai Jiaotong University School of Medicine, Shanghai, P.R. China

**Keywords:** breast cancer, HER2, older patients, trastuzumab, chemotherapy, multi-disciplinary treatment

## Abstract

**Objectives**: This study aims to evaluate influence factors for adjuvant chemotherapy regimen choice on the basis of trastuzumab in older human epidermal growth factor receptor 2 (HER2)-positive breast cancer under multi-disciplinary team (MDT) modality.

**Materials and Methods**: HER2-positive breast cancer patients aged ≥ 60 years who received breast cancer surgery between April 2013 and December 2017 in Shanghai Ruijin Hospital were retrospectively enrolled. Clinical and pathological features, MDT recommendations, administration of adjuvant treatment, cardiotoxicity, and disease outcome information were reviewed and analyzed.

**Results**: A total of 222 older HER2-positive breast cancer patients were included and recommended to receive adjuvant chemotherapy plus trastuzumab therapy. Paclitaxel plus trastuzumab (PH, 41/222, 18.5%), docetaxel plus carboplatin and trastuzumab (TCH, 62/222, 27.9%), and antharcyclines plus cyclophosphamide followed by taxanes and trastuzumab (AC-TH, 119/222, 53.6%) were the three main regimens. Patients with T1a-b (*P*<0.001), grade 1-2 (*P*=0.008), node-negative (*P*<0.001), stage I (*P*<0.001), low Ki-67 level (*P*<0.001) disease, with cardiovascular comorbidities (*P*=0.011), and aged ≥ 70 years (*P*<0.001) were more likely to be recommended to PH regimen. Among the 178 patients who finally received adjuvant chemotherapy plus one-year trastuzumab treatment, only four patients (4/117, 3.4%) were recorded to have asymptomatic LVEF declining ≥ 10% but remaining ≥ 50% within one-year trastuzumab treatment.

**Conclusions**: Clinical factors, including age, tumor size, node status, and cardiovascular comorbidity influenced the recommendation of trastuzumab with chemotherapy for older HER2-positive breast cancer patients. Low risk older HER2-positive breast cancer patients treated with PH had favorable outcome and good cardiac safety, which needed further clinical validation.

## Introduction

Human epidermal growth factor receptor 2 (HER2) is amplified and/or overexpressed in 15% to 20% breast cancers, which is associated with an aggressive phenotype and poor clinical outcome^1^-^4^. The prognosis of HER2 positive breast cancer has been improved dramatically with the application of anti-HER2 monoclonal antibody, trastuzumab^5^. One- year trastuzumab in combination with chemotherapy regimens, such as monotherapy paclitaxel (PH), docetaxel plus carboplatin (TCH) or doxorubicin plus cyclophosphamide followed by docetaxel (AC-TH), has been recognized as the standard of care for early HER2-positive breast cancer patients after the results from several prospective clinical trials^6^-^10^. Notably, in consideration of its adverse effects (AE), cardiotoxicity is the most common AE of trastuzumab treatment, especially among patients with older age or cardiovascular comorbidities^11^. However, older patients were underrepresented in pivotal clinical trials, and they were generally presented with comorbidities and may have more chance to increase treatment toxicity, especially cardiotoxicity, when they receive trastuzumab-based adjuvant treatment regimen. Several other studies investigated the patterns of chemotherapy, treatment duration, toxicity and the outcome of adjuvant trastuzumab in older patients, but they failed to specify the regimen of chemotherapy^12^-^15^.

Multi-disciplinary team (MDT) modality was firstly introduced in 1995 and now has been recognized as the standard management for breast cancer patients^16^-^17^. Breast oncologists and other healthcare professionals participate together in the MDT meeting and provide their personal treatment recommendation for each breast cancer patients. A final optimal regimen will be given after discussion which may improve patients' treatment compliance and survival^18^.

In this study, we focused on early HER2-positive breast cancer patients aged ≥ 60 years to explore influence factors for adjuvant chemotherapy regimen choice on the basis of trastuzumab treatment under MDT treatment modality. Treatment adherence to MDT recommendation, cardiotoxicity of trastuzumab, and prognostic outcomes were also analyzed in the enrolled patients.

## Materials and Methods

### Patients

Patients treated at Comprehensive Breast Health Center, Ruijin hospital from April 2013 to December 2017 were identified through Shanghai Jiaotong University Breast Cancer Database (SJTU-BCDB). Inclusion criteria were as follows: (1) Age ≥ 60 years; (2) Pathologically diagnosed as invasive breast cancer; (3) HER2-positive defined as HER2 3+ by immunohistochemical (IHC) or gene amplification by fluorescence *in situ* hybridization (FISH); and (4) Participating in MDT meeting. Patients who received neoadjuvant treatment were excluded. Clinicopathological characteristics, including age, surgery date, surgery type, tumor size stage, tumor grade, axillary lymph node status, estrogen receptor (ER) status, progesterone receptor (PR) status, Ki-67 index, and comorbidities, were reviewed and collected. Patient's MDT recommendation regimen and their actual adjuvant treatment regimens, LVEF value during one-year trastuzumab treatment, and follow-up information were also collected for further analysis. The study was approved by the Ethical Committees of Ruijin Hospital, Shanghai Jiaotong University School of Medicine. Additionally, a patient cohort was used to further explain our findings, which was extracted from Surveillance, Epidemiology, and End Results (SEER) database including patients ≥ 60 years with stage I-III HER2-positive early breast cancer treated during 2010-2015.

### Multi-disciplinary treatment system

MDT modality was adopted for routine breast cancer patient management in our center. Before MDT meeting, breast special nurse would provide patient's detailed demographic and clinicopathological information into the online MDT system (MDT4BC, http://rj.mdt.team:8080). Breast surgeons, medical oncologists, and radiologists then log into this MDT system and make the first-round vote for adjuvant treatment recommendation before MDT meeting. During MDT meeting, breast oncologists can review each physician's recommendation for individual patients and then make a discussion. After discussion, a second-round vote would be conducted and the final treatment decision was made for each patient.

### Survival

Disease-free survival (DFS) was defined as the time period from the date of disease diagnosis to the following events: local regional recurrence, distant recurrence, contralateral breast cancer, second primary cancer and death of any cause. We measured DFS as the months of disease-free from diagnosis through December 2018. Patients were censored at end of claims data in December 2018.

### Statistical methods

Chi-square test or Fisher's exact test was used to analyze the correlation between qualitative variables. Multinomial logistic regression model was used to examine the association between clinicopathological features and recommended treatment regimens. Kaplan-Meier curve and log-rank test were used to compare the survival among different treatment groups. All p values were two sided and less than 0.05 was considered statistically significant. IBM SPSS Statistics 22.0 and GraphPad Prism 7 were used for data analysis.

## Results

### Baseline clinical characteristics

**Figure 1:**
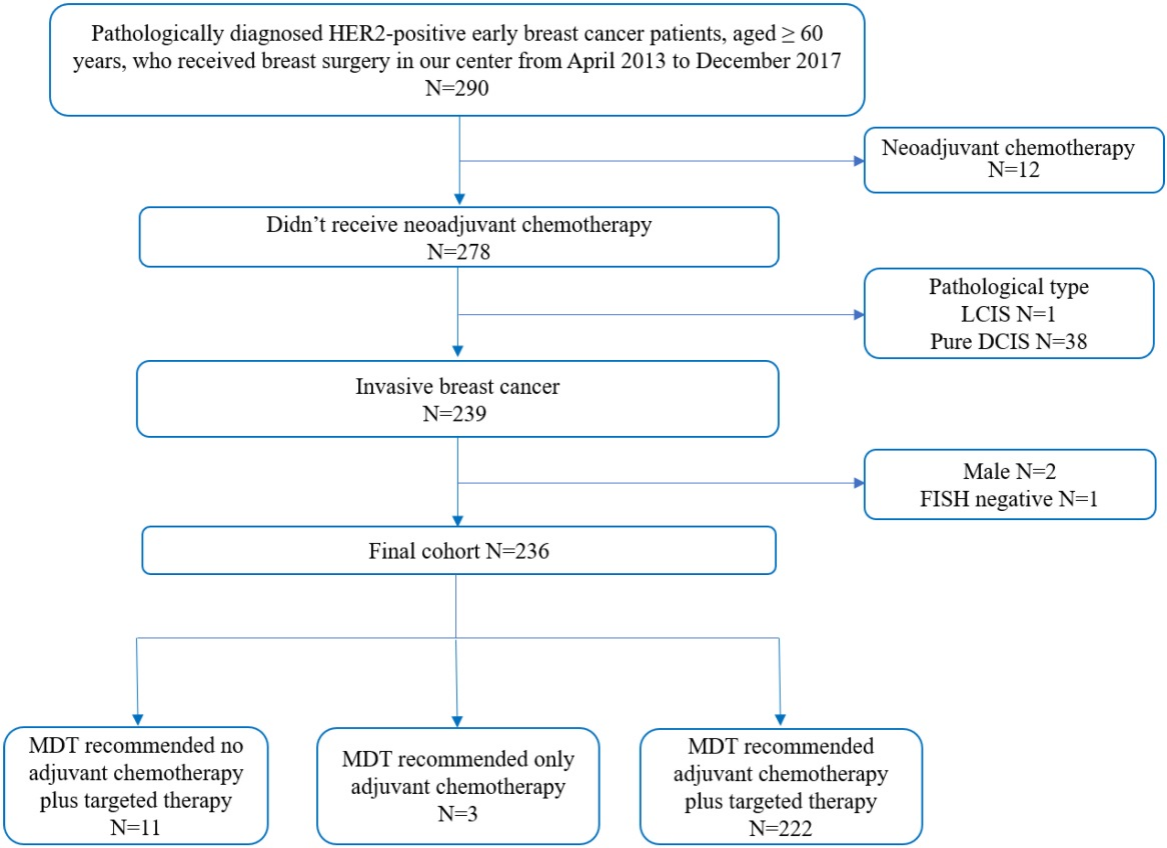
Enrollment diagram. HER2: human epidermal growth factor receptor 2; LCIS: lobular carcinoma in situ; DCIS: ductal carcinoma in situ; FISH: fluorescence in situ hybridization; MDT: multidisciplinary team.

Totally, 236 women met the enrollment criteria and 222 (94.0%) patients were recommended to receive adjuvant chemotherapy plus trastuzumab treatment under MDT meeting (Figure 1). There were six patients with bilateral breast cancer: four contralateral ductal carcinoma in situ and two luminal breast cancer. Since MDT discussion was conducted upon unilateral breast disease, clinicopathological features of HER2-positive disease were included for regimen recommendation analysis, while these six bilateral breast cancer patients were excluded for survival analysis. Baseline characteristics of the enrolled patients are shown in Table 1.

Median age was 64 years old (range, 60-95), and patients aged 60-70 years accounted for 81.5%. There were 82.9% patients receiving mastectomy and 55% patients treated with sentinel lymph node biopsy (SLNB). As for primary tumor size stage, 52.7% tumors were T2 and T3, and T1a-b and T1c disease accounted for 12.8% and 34.5%, respectively. In this study, 81 patients (36.8%) had axillary lymph node involvement and 125 patients (61.3%) had poor differentiation disease. According to the 7^th^ staging system of the American Joint Committee on Cancer (AJCC), 146 patients (66.7%) had stage II-III disease. A total of 95 patients (42.8%) had ER positive tumors and 60 patients (27.0%) had PR positive disease. The median Ki-67 index in this study was 40%, 199 patients (89.6%) had Ki-67 index ≥ 14%. When using the median Ki-67 (40%) as the cutoff level, 116 patients (52.3%) had high Ki-67 expression. Analyzing the body-mass index (BMI) of patients, 91 patients (41.0%) were overweight (BMI ≥ 24). Meanwhile, 112 patients (50.5%) had systemic comorbidities including hypertension, diabetes, coronary artery disease and valvular heart disease.

### Comparison of clinicopathological characteristics among different treatment recommendations

**Figure 2:**
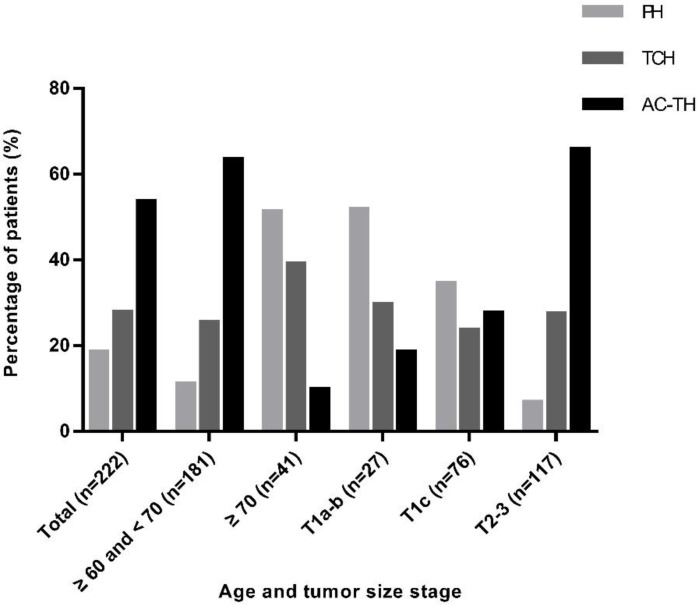
Adjuvant treatment regimen recommendation according to different age and tumor stage. Abbreviation: PH: paclitaxel plus trastuzumab; TCH: docetaxel plus carboplatin and trastuzumab; AC-TH: anthracyclines plus cyclophosphamide followed by taxanes and trastuzumab.

PH, TCH, and AC-TH regimens were recommended to 41 (18.5%), 62 (27.9%) and 119 (53.6%) patients respectively. Regarding PH regimen, patients with age ≥ 70 years (51.2%, *P*<0.001, Figure 2), T1a-b diseases (51.9%, *P*<0.001, Figure 2), grade I-II tumors (25.3%, *P*=0.008), negative axillary lymph node (26.6%, *P*<0.001), stage I (43.8%, *P*<0.001), and low Ki-67 level (25.5%, *P*<0.001) were greatly recommended. For patients who had systemic comorbidities, the recommendation rate for PH was higher than patients without comorbidities (23.2% vs 13.6%) while the percentage of recommending AC-TH was lower than patients without comorbidities (43.8% vs 63.6%) (*P*=0.011). Breast surgery procedure, histological subtype, ER and PR status and BMI had no significant influence on the choice of chemotherapy regimen (Table 1).

Multivariate logistic regression analysis demonstrated that the overall distribution of age, tumor size stage, ALN status, Ki-67 level and comorbidities were significantly different between PH, TCH and AC-TH regimen. Compared with AC-TH regimen, patients recommending to PH regimen were more often with older age (odds ratio [OR] 0.002, 95% confidence interval [CI] 0.000-0.017, *P*<0.001), small tumor size (OR 88.841, 95% CI 12.364-638.387, *P*<0.001), negative ALN (OR 0.007, 95% CI 0.001-0.084, *P*<0.001), low Ki-67 (OR 4.620, 95% CI 1.269-16.821, *P*=0.020), and with comorbidities (OR 2.960, 95% CI 0.865-10.129, *P*=0.084). Regarding the TCH regimen, patients with older age (OR 0.022, 95% CI 0.004-0.132, *P*<0.001), negative ALN status (OR 0.082, 95% CI 0.028-0.240, *P*<0.001), low Ki-67 level (OR 4.037, 95% CI 1.653-9.862, *P*=0.002) and comorbidities (OR 3.114, 95% CI 1.334-7.272, *P*=0.009) were more recommended than AC-TH regimen (Table 2).

### Patients' adherence to MDT recommendation

**Figure 3:**
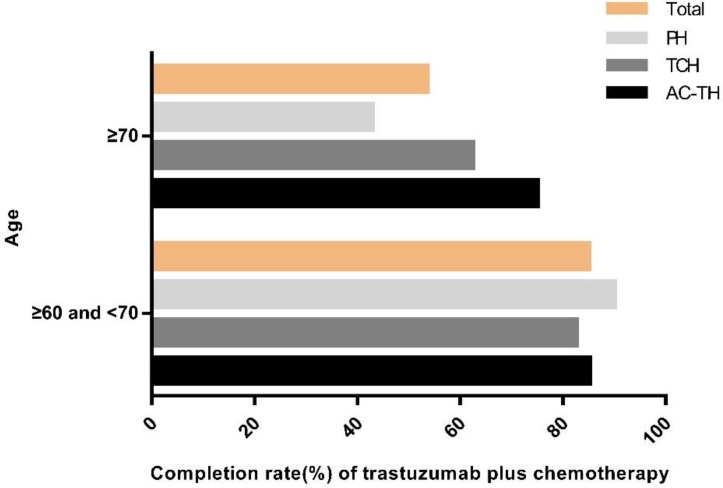
Completion rate of recommended regimens by age. Abbreviation: PH: paclitaxel plus trastuzumab; TCH: docetaxel plus carboplatin plus trastuzumab; AC-TH: anthracyclines plus cyclophosphamide followed by taxanes plus trastuzumab.

Adherence rates to MDT-recommending PH, TCH and AC-TH were 65.9% (27/41), 77.4% (48/62) and 84.9% (101/119), respectively. Patients who were recommended to AC-TH regimen had the highest completion rate. In the PH group, one patient (2.4%) received chemotherapy alone and 13 patients (31.7%) didn't receive trastuzumab or chemotherapy. Among 14 patients not compliant to TCH regimen, one patient (1.6%) received vinorelbine plus trastuzumab (NH) due to allergy to taxanes, another 6 patients (9.7%) received chemotherapy alone, one patient (1.6%) received trastuzumab alone, and the remaining 6 patients (9.7%) received neither trastuzumab nor chemotherapy. Furthermore, in the AC-TH group, 2 patients (1.7%) received TCH finally, 12 patients (10.1%) received chemotherapy alone and 4 patients (3.4%) received no trastuzumab or chemotherapy (Table 3). Patients with age ≥ 70 years were less likely than in the age 60-70 group (53.6% vs 85.1%, *P*<0.001) to complete adjuvant trastuzumab with chemotherapy, especially among PH regimen (42.9% vs 90.0%, *P*=0.003) (Figure 3).

### Cardiotoxicity

**Figure 4:**
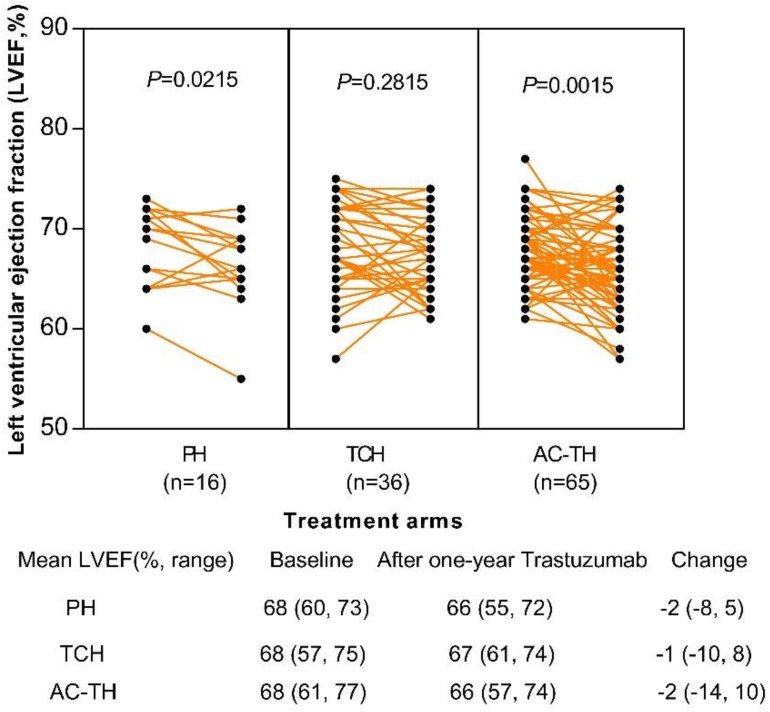
Change of left ventricular ejection fraction (LVEF) after one-year trastuzumab treatment among different treatment regimens. Abbreviation: PH: paclitaxel plus trastuzumab; TCH: docetaxel plus carboplatin plus trastuzumab; AC-TH: anthracyclines plus cyclophosphamide followed by taxanes plus trastuzumab.

Totally 117 patients' echocardiogram results of baseline and after one-year trastuzumab administration were obtained: 16 (13.7%) in the PH, 36 (30.8%) in the TCH, and 65 (55.5%) in the AC-TH treatment group, respectively. After one-year administration of trastuzumab, left ventricular ejection fraction (LVEF) changed significantly among patients receiving PH regimen (*P*=0.022) or AC-TH regimen (*P*=0.002), but no statistically significant change in the TCH regimen (*P*=0.282). However, only 4 (3.4%) patients experienced LVEF declining ≥ 10%: one in the TCH group, and other three in the AC-TH group. None of patients had LVEF decline less than 50%. The mean LVEF change after one-year trastuzumab were -2 (-8, 5), -1 (-10, 8), and -2 (-14, 10) in the PH, TCH and AC-TH groups (Figure 4).

### Disease outcomes

**Figure 5:**
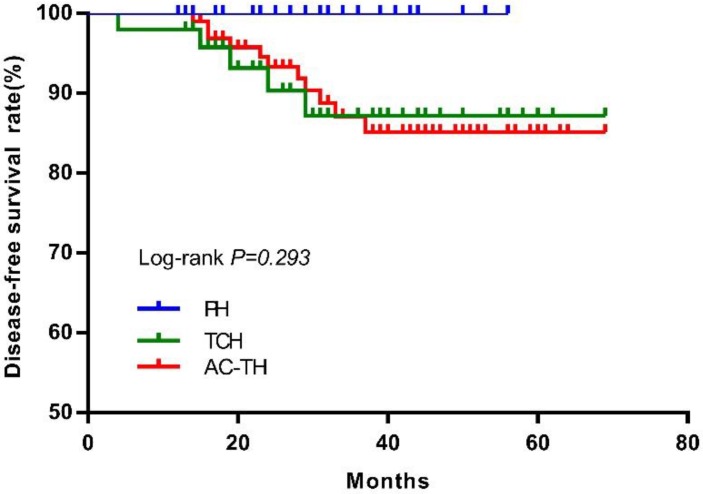
Disease-free survival according to different treatment regimen. Abbreviation: PH: paclitaxel plus trastuzumab; TCH: docetaxel plus carboplatin plus trastuzumab; AC-TH: anthracyclines plus cyclophosphamide followed by taxanes plus trastuzumab.

After excluding 4 bilateral breast cancer patients, there were 174 patients who finally received PH (n=27), TCH (n=49) or AC-TH (n=98) treatment. Sixteen DFS events were recorded after a median follow-up of 32 months (range, 4-69), including 6 distant recurrences, 2 local recurrences, one contralateral breast cancer, 6 second primary cancer and one death. The estimated 3-year DFS rates were 100.0%, 87.2%, and 87.0% for patients treated with PH, TCH, or AC-TH respectively (*P*= 0.293, Figure 5).

### Results from SEER database

Patients' baseline characteristics in the population-cohort extracted from SEER database were shown in Table S1. Among the 13621 patients, about 42% patients ≥ 70 years old with a median age of 68. There were 54.1% women had T1 tumor, and 34.1% patients had lymph node involvement. A total of 9425 (69.2%) patients had ER positive disease. Adjuvant treatment information indicated that 8663 patients received chemotherapy while other 4958 were not. Both univariate and multivariate analysis demonstrated that age, tumor size, tumor grade, lymph node involvement, ER and PR status influenced chemotherapy administration (P <0.001; Table S1 and Table S2). Patients with young age, large tumors, lymph node metastasis and negative hormone receptor tended to receive chemotherapy. After a median follow-up of 38 months, there were 1638 patients died, and chemotherapy administration was significant associated with superior overall survival (OS) (P<0.001, Figure S1).

## Discussion

In current study, we found that 94.0% HER2-positive older breast cancer patients were suggested to receive trastuzumab with chemotherapy under MDT discussion, which was higher than previous studies (50%-85.2%)^19^-^20^. Not surprisingly, we also demonstrated that clinical features such as age, tumor size, nodal status, Ki-67 level, and comorbidity influenced chemotherapy regimen choice in the basis of trastuzumab treatment. HER2+ breast cancer patients with high risk factors were more likely to be recommended with AC-TH treatment (53.6%) than those treated with PH (18.5%) or TCH (27.9%). Results from SEER database also demonstrated that patients with relatively young age, large tumor, lymph node involvement, and ER-negative disease were more likely to receive chemotherapy. Because SEER database did not provide specific chemotherapy regimen and treatment information of trastuzumab, we just simply analyzed influencing factors of chemotherapy administration and its association with outcome. Patients who did not receive chemotherapy had significantly worse OS than patients who received. Therefore, further studies were in need to explore optimal treatments for older HER2-positive patients to improve prognosis.

Trastuzumab concurrent with anthracycline-taxane based regimens were well established in high risk HER2-positive breast cancer patients. However, few data were available regarding adjuvant trastuzumab treatment choice in older breast cancer patients, especially for those with small, node-negative, and HER2-positive disease. Previous studies found that patients with pT1a-bN0, HER2-positive breast cancer had worse disease outcome than HER2-negative diseases^21^-^23^, which may indicate potential benefit from adjuvant trastuzumab treatment^24^-^25^. The APT trial demonstrated that patients with tumor size ≤ 3.0 cm, node-negative HER2-positive breast cancer had a favorable 3-year invasive disease-free survival (iDFS) of 98.7% treated with weekly paclitaxel and trastuzumab, including 33.7% patients ≥ 60 years and 10.1% patients ≥ 70 years old 26. Therefore, older HER2-positive breast cancer patients even with relative low risk can also benefit from trastuzumab treatment, indicating PH regimen is an optional choice. In our present study, we enrolled 236 HER2-positive breast cancer patients with age ≥ 60 years disease and patients with aged ≥ 70 years (21/41, 51.2%) or T1a-b tumors (14/27, 51.9%) were more likely to be recommended to receive PH treatment. Relatively short follow up period found PH regimen was well tolerated (none LVEF declining ≥10%) and was associated with favorable disease outcome (3-year DFS 100%).

Completion rate of older HER2+ breast cancer patients received trastuzumab treatment in this study was 80.2%, which was higher than patients in the SEER-Medicare database (reported as 40%-52%)^19^. Our study found that age and treatment regimen were associated with patients' treatment adherence. Patients aged 60-70 years were associated with significantly higher adherence rate than patients aged ≥70 years (85.1% vs 53.6%, *P*<0.001), especially in the PH regimen. Other factors, including side effect, poor socioeconomic status, and low educational level, can also explain the nonadherence of treatment in older breast cancer patients.

Older breast cancer patients generally presented with more comorbidities and may have more probabilities to experience treatment toxicity, especially receiving trastuzumab plus chemotherapy. Comparing patients' baseline and one-year trastuzumab treatment in terms of echocardiogram, we found older patients were generally well tolerated for cardiotoxicity. A study used SEER-Medicare data also reported that AC-TH was not associated with a higher rate of serious adverse events than TCH regimen^14^, indicating anthracycline-taxane based chemotherapy plus trastuzumab (AC-TH) is also a reasonable and safe regimen for high risk older HER2+ breast cancer patients.

DFS rate of patients who receiving PH regimen was better than the other two regimens in this study, but there were no significantly difference (*P*=0.293). Patients receiving AC-TH regimen had more high-risk tumors (large tumor size, ALN metastasis, and grade III disease) may explain this phenomenon. Since pivotal adjuvant trials established the backbone efficacy of AC-TH and TCH regimens for high-risk HER2-positive breast cancer, which enrolled about 15% patients older than 60 years 6-10. The APT trial established the role of PH regimen for women with small, node-negative, HER2-positive breast cancer (33.7% patients older than 60 years). Therefore, AC-TH, TCH, or PH regimens could be all considered as optimal adjuvant treatment regimens for older HER2+ breast cancer patients. However, appropriate treatment for patients older than age 70 or with significant comorbidities is still unclear. Considering the incidence of breast cancer is associated with aging and number of older breast cancer patients will increase for its long life expectancy^15^,^27^. Optimal treatment with less toxicity for these population needs further exploring.

Several potential limitations may exist in this study. First, this is a single-center retrospective study with relatively small sample size and the results couldn't be entirely validated with an independent population-based cohort from public database. But our study innovatively analyzed specific chemotherapy regimens in combination with MDT modality. Second, only 117 of 178 patients' (66.5%) echocardiogram results were collected during one-year trastuzumab treatment, which can't comprehensively evaluate the actual cardiac safety of these three regimens. Moreover, our study was underpowered to detect prognostic difference among different treatment groups due to its relative short follow up period and few recurrence events. Further exploration of treatment toxicities and outcomes for older patients with HER2-positive breast cancer is warranted.

In conclusion, our study found that the majority of older HER2-positive breast cancer patients would be recommended to receive adjuvant trastuzumab and chemotherapy. Patients with small tumor size, low grade, node-negative, low Ki-67, age ≥ 70 years, and comorbidities were more likely to receive PH treatment. Trastuzumab in combination with chemotherapy was well-tolerated with good cardiac safety and superior disease outcome among older breast cancer patients, which needs further clinical evaluation.

## Supplementary Material

Supplementary figures and tables.

## Figures and Tables

**Table 1:** Clinicopathological characteristics and treatment regimens of enrolled patients.

Characteristics	Total (n=222) No. (%)	PH (N=41) No. (%)	TCH (n=62) No. (%)	AC-TH (n=119) No. (%)	P value
**Age (median, range)**	64 (60, 95)				<0.001
≥ 60 and < 70	181 (81.5)	20 (11.0)	46 (25.4)	115 (63.5)	
≥ 70	41 (18.5)	21 (51.2)	16 (39.0)	4 (9.8)	
**Breast surgery**					0.797
Breast-conserving	38 (17.1)	7 (18.4)	9 (23.7)	22 (57.9)	
Mastectomy	184 (82.9)	34 (18.5)	53 (28.8)	97 (52.7)	
**ALN surgery**					<0.001
SLNB	121 (55.0)	34 (28.1)	42 (34.7)	45 (37.2)	
ALND	99 (45.0)	5 (5.1)	20 (20.2)	74 (74.7)	
No	2	2			
**Histological subtype**					0.550
IDC	204 (91.9)	36 (17.6)	58 (28.4)	110 (53.9)	
Non-IDC	18 (8.1)	5 (27.8)	4 (22.2)	9 (50.0)	
**Tumor stage**					<0.001
T1a-b	27 (12.8)	14 (51.9)	8 (29.6)	5 (18.5)	
T1c	76 (34.5)	18 (23.7)	21 (27.6)	37 (48.7)	
T2-3	117 (52.7)	8 (6.8)	32 (27.4)	77 (65.8)	
Tx	2	1	1		
**Tumor grade**					0.008
I-II	79 (38.7)	20 (25.3)	26 (32.9)	33 (41.8)	
III	125 (61.3)	15 (12.0)	32 (25.6)	78 (62.4)	
Unknown	18	6	4	8	
**ALN status**					<0.001
Negative	139 (63.2)	37 (26.6)	48 (34.5)	54 (38.8)	
Positive	81 (36.8)	2 (2.5)	14 (17.3)	65 (80.2)	
Nx	2	2			
**Stage**					<0.001
I	73(33.3)	32(43.8)	23(31.5)	18(24.7)	
II	107(48.9)	7(6.5)	33(30.8)	67(62.6)	
III	39(17.8)	0	5(12.8)	34(87.2)	
Unknown	3				
**ER status**					0.273
Positive	95 (42.8)	20 (21.1)	30 (31.6)	45 (47.4)	
Negative	127 (57.2)	21 (16.5)	32 (25.2)	74 (58.3)	
**PR status**					0.617
Positive	60 (27.0)	11 (18.3)	14 (23.3)	35 (58.3)	
Negative	162 (73.0)	30 (18.5)	48 (29.6)	84 (51.9)	
**Ki-67, %**					<0.001
< 40	106 (47.7)	27 (25.5)	37 (34.9)	42 (39.6)	
≥ 40	116 (52.3)	14 (12.1)	25 (21.6)	77 (66.4)	
**BMI**					0.487
Normal (<24)	131 (59.0)	21 (16.0)	39 (29.8)	71 (54.2)	
Overweight (≥24)	91 (41.0)	20 (22.0)	23 (25.3)	48 (52.7)	
**Comorbidity**					0.011
No	110 (49.5)	15 (13.6)	25 (22.7)	70 (63.6)	
Yes	112 (50.5)	26 (23.2)	37 (33.0)	49 (43.8)	

Abbreviation: PH: paclitaxel plus trastuzumab; TCH: docetaxel plus carboplatin and trastuzumab; AC-TH: anthracyclines plus cyclophosphamide followed by taxanes and trastuzumab; ALN: axillary lymph node; SLNB, sentinel lymph node biopsy; ALND: axillary lymph node dissection; IDC: invasive ductal carcinoma; Non-IDC: non-invasive ductal carcinoma; ER: estrogen receptor; PR: progesterone receptor. The median Ki-67 index in this study was 40%, therefore, it was used as the cutoff to define low and high Ki-67 expression. Comorbidity includes hypertension, diabetes, coronary artery disease and valvular heart disease.

**Table 2:** Multivariate analysis of the correlation between clinicopathological characteristics and treatment regimens ^a^.

Characteristics	PH				TCH			*P* value
	OR	95%CI	*P* value		OR	95%CI	*P* value	
**Age (years)**								<0.001
≥ 60 and < 70	0.002	0.000-0.017	<0.001		0.022	0.004-0.132	<0.001	
≥ 70	Reference				Reference			
**Tumor size stage**								<0.001
T1a-b	88.841	12.364-638.387	<0.001		3.830	0.934-15.710	0.062	
T1c	7.516	1.609-35.116	0.010		1.877	0.769-4.585	0.167	
T2-3	Reference				Reference			
**Tumor grade**								0.248
I-II	2.819	0.809-9.819	0.104		1.312	0.540-3.191	0.549	
III	Reference				Reference			
**ALN status**								<0.001
Positive	0.007	0.001-0.084	<0.001		0.082	0.028-0.240	<0.001	
Negative	Reference				Reference			
**Ki-67 index**								0.004
< 40	4.620	1.269-16.821	0.020		4.037	1.653-9.862	0.002	
≥ 40	Reference				Reference			
**Comorbidity**								0.023
Yes	2.960	0.865-10.129	0.084		3.114	1.334-7.272	0.009	
No	Reference				Reference			

Abbreviation: PH: paclitaxel plus trastuzumab; TCH: docetaxel plus carboplatin and trastuzumab; AC-TH: anthracyclines plus cyclophosphamide followed by taxanes and trastuzumab; ALN: axillary lymph node; OR: odds ratio; 95% CI: 95% confidence interval. ^a^The reference category for recommended treatment regimen is AC-TH.

**Table 3:** Treatment compliance to MDT recommendation.

MDT recommendation	No.	Actual received regimens No. (%)						
		PH	TCH	AC-TH	Other	Only Chemo	Only Tras	No treatment
PH	41	27 (65.9)				1 (2.4)		13 (31.7)
TCH	62		48 (77.4)		1 (1.6)	6 (9.7)	1 (1.6)	6 (9.7)
AC-TH	119		2 (1.7)	101 (84.9)		12 (10.1)		4 (3.4)

Abbreviation: MDT: multidisciplinary treatment; Chemo: chemotherapy; PH: paclitaxel plus trastuzumab; TCH: docetaxel plus carboplatin and trastuzumab; AC-TH: anthracyclines plus cyclophosphamide followed by taxanes and trastuzumab; Other regimen included one patient in TCH group received vinorelbine plus trastuzumab (NH) due to allergic to taxanes; Tras: trastuzumab.
