# Supplementary material for: Factors Influencing Adjuvant Chemotherapy and Trastuzumab Choice in Older Human Epidermal Growth Factor Receptor 2-positive Breast Cancer Patients
Source: J Cancer. 2020 Feb 14;11(9):2602–9. doi: 10.7150/jca.39509 (PMC7066021; doi:10.7150/jca.39509)
Supplement: Supplementary file 1 — Supplementary figures and tables. [file jcav11p2602s1.pdf]

Table S1. Baseline characteristics of enrolled patients from SEER database.

**Characteristics**	**Total (n=13621) No. (%)**	**Chemotherapy (n=8663) No. (%)**	**No/Unknown chemotherapy (n=4958) No. (%)**	**P value**
**Age (median, range)**	68 (60, 101)			<0.001
≥ 60 and < 70	7908 (58.1)	5916 (74.8)	1992 (25.2)	
≥ 70	5713 (41.9)	2747 (48.1)	2966 (51.9)	
**Race**				0.084
Hispanic/(All/Races)	1256 (9.2)	788 (62.7)	468 (37.3)	
Non-Hispanic/White	9695 (71.2)	6148 (63.4)	3547 (36.6)	
Non-Hispanic/Asian/or/Pacific/Islander	1176 (8.6)	736 (62.6)	440 (37.4)	
Non-Hispanic/Black	1449 (10.6)	966 (66.7)	483 (33.3)	
Non-Hispanic/Unknown/Race	45 (0.3)	25 (55.6)	20 (44.4)	
**Breast surgery**				0.094
Breast-conserving	7321 (53.7)	4608 (62.9)	2713 (37.1)	
Mastectomy	6285 (46.1)	4043 (64.3)	2242 (35.7)	
Unknown	15 (0.1)			
**Histological subtype**				0.135
IDC	12504 (91.8)	7980 (63.8)	4524 (36.2)	
ILC	591 (4.3)	354 (59.9)	237 (40.1)	
IDC and ILC	526 (3.9)	329 (62.5)	197 (37.5)	
**Tumor stage**				<0.001
T1a-b	3027 (22.2)	1234 (40.8)	1793 (59.2)	
T1c	4345 (31.9)	2910 (67.0)	1435 (33.0)	
T2	4921 (36.1)	3507 (71.3)	1414 (28.7)	
T3	754 (5.5)	580 (76.9)	174 (23.1)	
T4	567 (4.2)	427 (75.3)	140 (24.7)	
Tx	7 (0.1)			
**Tumor grade**				<0.001
I	807 (5.9)	338 (41.9)	469 (58.1)	
II	5003 (36.7)	2966 (59.3)	2037 (40.7)	

III-IV	7318 (53.7)	5107 (69.8)	2211 (30.2)	
Unknown	493 (3.6)	252 (51.1)	241 (48.9)	
**ALN status**				<0.001
Negative	8977 (65.9)	5066 (56.4)	3911 (43.6)	
Positive	4639 (34.1)	3595 (77.5)	1044 (22.5)	
Nx	5			
**Stage**				<0.001
I	6259 (46.0)	3247 (51.9)	3012 (48.1)	
II	5337 (39.2)	3838 (71.9)	1499 (28.1)	
III	2025 (14.9)	1578 (77.9)	447 (22.1)	
**ER status**				<0.001
Positive	9425 (69.2)	5762 (61.1)	3633 (38.9)	
Negative	4195 (30.8)	2901 (69.2)	1294 (30.8)	
Unknown	1			
**PR status**				<0.001
Positive	6951 (51.0)	4190 (60.3)	2761 (39.7)	
Negative	6651 (48.8)	4459 (67.0)	2192 (33.0)	
Unknown	19 (0.1)			
**Death**				<0.001
Dead	1638 (12.0)	714 (43.6)	924 (56.4)	
Alive	11983 (88.0)	7949 (66.3)	4034 (33.7)	

Abbreviation: IDC: Infiltrating duct carcinoma; ILC: Infiltrating lobular carcinoma; Infiltrating duct and lobular carcinoma; IDC and ILC: ALN: axillary lymph node; ER: estrogen receptor; PR: progesterone receptor.

Table S2. Multivariate analysis of the correlation between clinicopathological characteristics and chemotherapy recommendation using SEER data [a].

Characteristics	OR	95%CI	*P* value
**Age (years)**			<0.001
≥ 60 and < 70	4.019	3.706-4.360	
≥ 70	Reference		
**Tumor stage**			<0.001
T1a-b	0.586	0.428-0.803	
T1c	1.685	1.241-2.289	
T2	0.940	0.726-1.219	
T3	1.115	0.841-1.479	
T4	Reference		
**Tumor grade**			<0.001
I	0.461	0.389-0.545	
II	0.782	0.718-0.852	
III	Reference		
**ALN status**			<0.001
Positive	0.650	0.573-0.737	
Negative	Reference		
**Stage**			0.107
I	0.426	0.322-0.565	
II	0.909	0.760-1.087	
III	Reference		
**ER status**			0.001
Positive	0.821	0.736-0.917	
Negative	Reference		
**PR status**			0.009
Positive	0.866	0.784-0.955	
Negative	Reference		

Abbreviation: ALN: axillary lymph node; ER: estrogen receptor; PR: progesterone receptor; OR: odds ratio; 95% CI: 95% confidence interval.

[a]The reference category of chemotherapy information was No/Unknown.

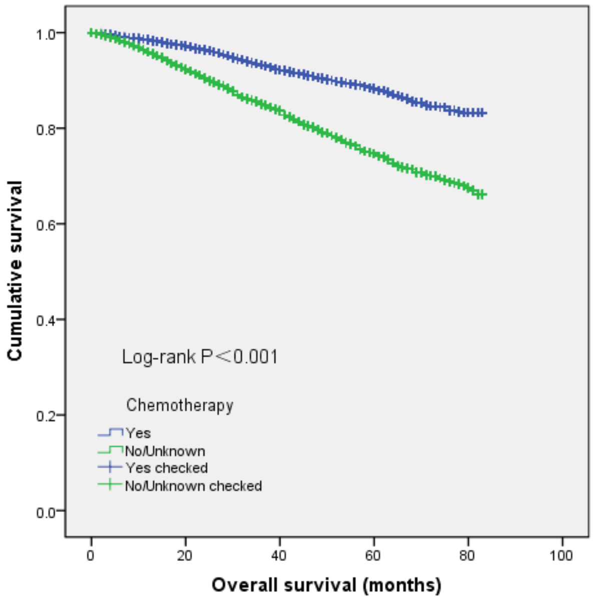

Figure S1. Overall survival of patients received chemotherapy or not from SEER data.
